# Improved YOLOv4-tiny network for real-time electronic component detection

**DOI:** 10.1038/s41598-021-02225-y

**Published:** 2021-11-23

**Authors:** Ce Guo, Xiao-ling Lv, Yan Zhang, Ming-lu Zhang

**Affiliations:** School of Mechanical Engineerings, Hebei University of Technology, Tianjin, 300131 China

**Keywords:** Electrical and electronic engineering, Computer science

## Abstract

In the electronics industry environment, rapid recognition of objects to be grasped from digital images is essential for visual guidance of intelligent robots. However, electronic components have a small size, are difficult to distinguish, and are in motion on a conveyor belt, making target detection more difficult. For this reason, the YOLOv4-tiny method is used to detect electronic components and is improved. Then, different network structures are built for the adaptive integration of middle- and high-level features to address the phenomenon in which the original algorithm integrates all feature information indiscriminately. The method is deployed on an electronic component dataset for validation. Experimental results show that the accuracy of the original algorithm is improved from 93.74 to 98.6%. Compared with other current mainstream algorithms, such as Faster RCNN, SSD, RefineDet, EfficientDet, and YOLOv4, the method can maintain high detection accuracy at the fastest speed. The method can provide a technical reference for the development of manufacturing robots in the electronics industry.

## Introduction

As a long-standing hot issue in the field of computer vision, target detection aims to determine the class and location of a specific object in an image. It is widely used in various autonomous intelligent systems to provide key information for subsequent intelligent navigation, security alarms, motion planning, and other decisions, such as autonomous driving^1^, security monitoring^2^, drone navigation^3^, and industrial robots^4^. Deep learning algorithms have achieved the best performance in the field of target detection and can be broadly classified into two-stage and one-stage detection algorithms. However, two-stage algorithms are too slow and therefore perform poorly in applications. By contrast, accuracy is the key measure to improve the speed of one-stage algorithms. Many intelligent systems have high real-time requirements for target detection. For example, intelligent robots need to identify targets in real time to complete avoidance or grasping actions; unmanned aerial vehicle navigation needs to identify targets in remote sensing images in real time to complete subsequent decisions; and autonomous driving needs to identify targets, such as traffic lights, road signs, and pedestrians, in real time to ensure safe driving.

In this research, we study the detection of electronic components by robots in the electronics industry. The robots collect image data by using charge-coupled device (CCD) industrial cameras, determine the class and location of the object from digital images, and realize 3D trajectory planning. Real-time detection of the target object is one of the most challenging tasks. We simulate the electronic industrial environment and use CCD industrial cameras to capture images of electronic components on a conveyor belt and perform electronic component detection. Given the limitation of computing resources in the industrial environment and real-time considerations, we deploy YOLOv4-tiny^5^ in a simulated industrial environment. The original algorithm is designed on the basis of a generic target, and simply applying the existing detection model may lead to poor performance. First, to address the problem of high similarity between various types of electronic components, an improved cross-entropy function is used to replace the original classification loss part and increase the proportion of hard-to-classify samples in the training. Second, the information of different feature layers is integrated with a learnable approach. Related studies^6^ have shown that middle-layer features contain rich spatial and semantic information, and higher-layer features contain richer semantic information. Thus, different network structures are designed for different layers for information integration, and feature redundancy is reduced.

The contributions of this study are as follows.

The electronic industry environment is simulated, and an electronic component dataset is established. The YOLOv4-tiny detector is transplanted to the field of robotics in the electronics industry instead of the traditional method, thus providing a technical reference for the development of related robots.

In consideration of practical application scenarios, the YOLOv4-tiny algorithm is improved from two perspectives. One is from the perspective of the loss function so that the hard-to-classify samples can be trained more adequately. The second one applies different attention mechanisms to middle-level features and high-level features from the perspective of feature layer.

The improved method is experimentally validated on an electronic component dataset and achieves a large improvement in accuracy compared with the original method. We compare the proposed method with current state-of-the-art target detection methods and demonstrate its superiority and effectiveness in the domain of electronic components detection.

The rest of the paper is organized as follows: “Related work” briefly summarizes the existing work related to our task. Section “Methods” describes our approach in detail. Section “Experimental results and discussion” shows and analyzes the experimental results of our method after its deployment. Section “Conclusion” concludes the paper.

## Related work

Traditional target detection methods use manual annotation of features and build shallow detectors. Early target detection methods originated from face recognition, and the earliest method is the Viola–Jones^7^ algorithm. It establishes an integral image, uses the image to obtain several different rectangular features quickly, and completes face recognition through a cascade of classifiers; however, the detection of side face images is unstable. Different feature point representations, such as SIFT^8^ and HOG^9^, have been explored in subsequent studies; these representations are robust to geometric and optical variations. DPM^10^ detects a wide range of complex objects. Although these traditional methods perform well in some specific scenes, they perform substantially worse in differential variations of different backgrounds. Thus, a hierarchical, multistage process is needed to compute features that are more informative for visual recognition.

Deep learning algorithms have gradually replaced traditional algorithms for the manual labeling of features by automatically learning features through convolutional neural networks (CNNs). The regional CNN (R-CNN)^11^ proposed by Girshick et al. is the earliest two-stage algorithm that uses selective search methods to generate candidate regional networks and uses CNNs to extract features fed into classifiers. In 2014, He et al. proposed SPPNet^12^, which uses different sizes of convolution kernels. Girshick et al. proposed Fast R-CNN^13^, replacing the last layer with the pooling layer of interest. Faster R-CNN^14^ proposed by Ren et al. uses anchor boxes of different scales to extract features, reducing the generation time of candidate boxes. Faster R-CNN achieved 78.8% mean average precision (mAP) when running at 5 frames per second (FPS) on PASCAL VOC2007.

The two-stage algorithm has high detection accuracy and has achieved success in the pursuit of ultra-high detection accuracy. However, the candidate frame generation process entails a large amount of calculation, resulting in insufficient detection efficiency. Therefore, numerous one-stage algorithms, such as YOLO^15^, SSD^16^, RefineDet^17^, EfficientDet^18^, and other methods, have been investigated in recent years. The YOLO algorithm has shown superior performance and has been updated from YOLOv2 to YOLOv4 after several optimizations^19–21^. In consideration of mobile application requirements, a more simplified version of Tiny was proposed^5^. The single-stage detection method significantly improves the computational efficiency and meets real-time requirements, but its accuracy can still be improved. In the next section, we analyze the limitations of YOLO for electronic component testing and the optimizations it has undergone.

## Methods

### Visual system and our visual detection tasks

**Figure 1:**
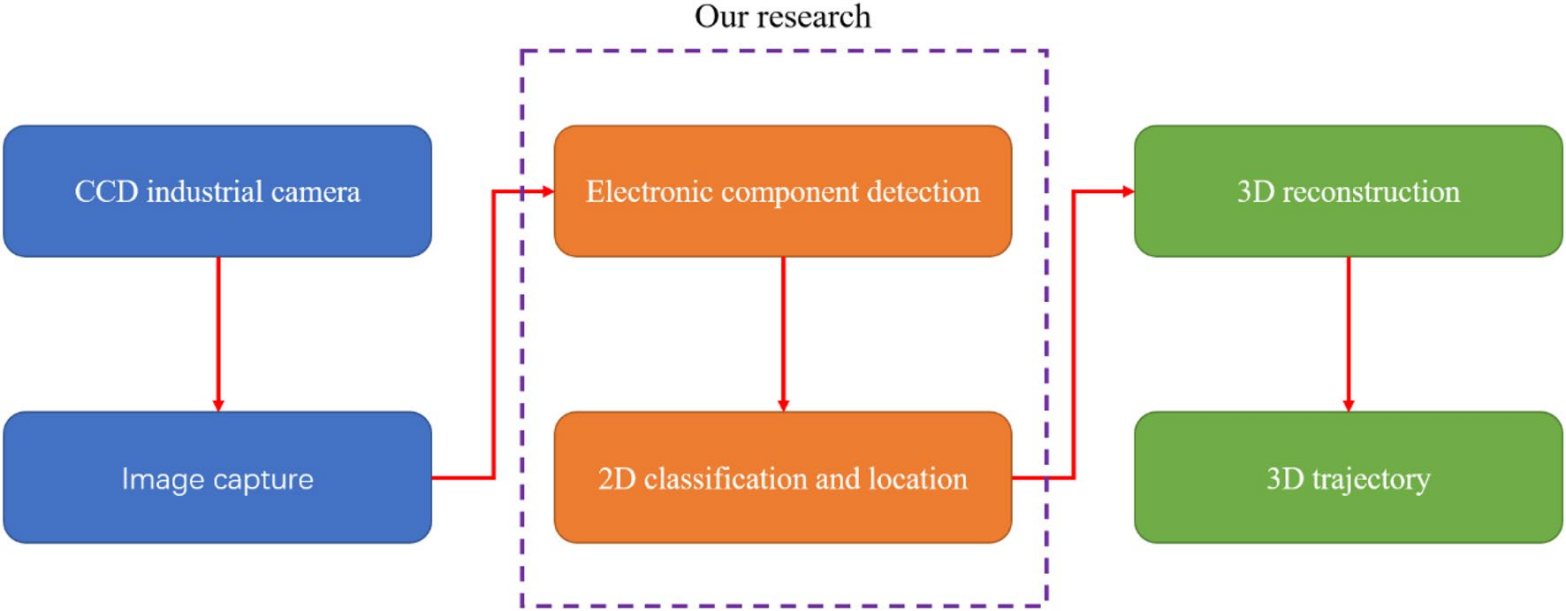
Robot vision system in the electronics manufacturing industry. First, a CCD industrial camera is used to collect images of electronic components, then algorithms are used to perform two-dimensional detection of electronic components. Lastly, three-dimensional reconstruction of the obtained two-dimensional classification and positioning information is performed to obtain the corresponding trajectory.

This case is applied to the visual guidance of a manufacturing robot in the electronics industry. The robot is composed of vision, control, and mechanical systems. The vision system is responsible for the real-time identification and positioning of the electronic components on the industrial conveyor belt. The system process is shown in Fig. 1. The information obtained from 2D electronic component detection will be used for 3D reconstruction. The electronic components move with the industrial conveyor belt; thus, the three-dimensional trajectory of the components needs to be obtained to complete the grasping action. This feature is the basis for dynamic planning of the manipulator and correction of movement. Classification and positioning information are sent to the control system, which finally guides the mechanical system to perform the gripping action. The vision system is the most critical and challenging part of the robot because it provides the basis for subsequent control of the robot. In the remainder of this paper, we focus on 2D electronic component detection, which is the core task of the vision system.

The vision system needs to ensure that the images captured by the camera can be analyzed in real time. Therefore, the YOLO detector is suitable. Considering the high real-time requirements, we choose the latest YOLOv4-tiny detector as the baseline model of the electronic component detector. Although YOLOv4-tiny can already detect the input image in real time, the detection accuracy is insufficient. In this case, the dataset contains electronic components of different sizes, and small parts have a small proportion in the training. In addition, electronic components are different from general datasets, with low discrimination between parts and a high false detection rate. Therefore, we enhance the detector to accommodate the need for accurate real-time detection of two-dimensional images. The remainder of this section focuses on these improvement methods.

### Improvement of the loss function

The YOLOv4-tiny model minimizes the loss function through parameter iterative regression to achieve the effect of model optimization. The loss function is divided into three parts, namely, classification loss, confidence loss, and complete intersection over union (CIOU) loss. Classification loss is used to fit object category information; confidence loss is used to determine whether the target is included; and CIOU loss is composed of the overlap area, center point distance, and aspect ratio, and is used to fit positioning information. In consideration of the high similarity between the various categories of electronic components and the stable aspect ratio, the loss function in YOLOv4-tiny can be modified accordingly to improve the training effect. The following content details the specific modifications.

In the classification loss, YOLOv4-tiny predicts a probability value for each category. We use $\widehat{P}$ to represent the predicted value. Through continuous fitting with the true value *P* (0 or 1, representing whether it is the category), the prediction effect is obtained. Given that each category can be viewed as a binary classification problem, YOLOv4-tiny uses the binary cross-entropy (BCE) loss commonly used for binary classification with the following equation:

1 $$BCE\left( {\hat{P},P} \right) = \left\{ {\begin{array}{*{20}l} { - \log \;\hat{P}} \hfill & {if\;P = 1} \hfill \\ { - \log \left( {1 - \hat{P}} \right)} \hfill & {otherwise} \hfill \\ \end{array} } \right..$$

**Figure 2:**
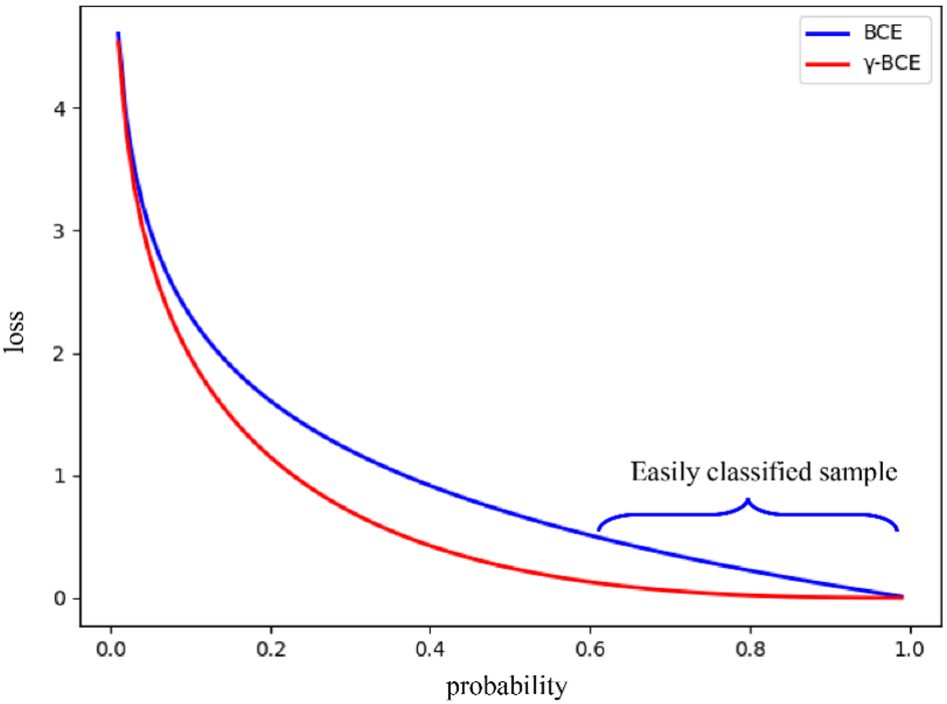
BCE curve comparison before and after improvement.

We analyze the BCE curve, and the results are presented in Fig. 2. To simplify the curve, we define $Px$ as

2 $$Px = \left\{ {\begin{array}{*{20}l} {\hat{P}} \hfill & {if\;P = 1} \hfill \\ {1 - \hat{P}} \hfill & {otherwise} \hfill \\ \end{array} } \right..$$

Then, $$BCE(Px)=-\mathrm{log}Px$$.

To reduce the proportion of easily classified samples in the loss function, we refer to the research by Focal Loss^22^ on general target detection and add a modulation term on the basis of the original cross-entropy function named γ-BCE. The binary cross-entropy function is replaced as follows.

3 $$BCE\left(Px\right)=-{\left(1-Px\right)}^{\gamma }*\mathrm{log}Px.$$

We take accuracy as the standard and finally determine through experiments that the value of γ is 1.5 in this task, which is explained in detail in “Experimental results and discussion”. We draw the image of γ-BCE in Fig. 2 and compare it with the original image. The result is shown in Fig. 2.

t, S × S grid multiobjective classification has classes, and we classify the loss function as

4 $${L}_{class}=-{\sum }_{i=0}^{S*S}{1}_{ij}^{obj}\sum_{t\in classes}\left[{{\left({\widehat{P}}_{i}\left(t\right)\right)}^{\gamma }P}_{i}\left(t\right)\mathrm{log}{\widehat{P}}_{i}\left(t\right)+{\left(1-{\widehat{P}}_{i}\left(t\right)\right)}^{\gamma }\left(1-{\widehat{P}}_{i}\left(t\right)\right)\mathrm{log}\left(1-{\widehat{P}}_{i}\left(t\right)\right)\right].$$

The CIOU loss in YOLOv4-tiny consists of three parts, namely, the overlap area, center point distance, and aspect ratio, as follows:

5 $${L}_{ciou}=1-IoU+{\rho }^{2}\left(b,{b}^{gt}\right)/{c}^{2}+{v}^{2}/\left[\left(1-Iou\right)+v\right],$$

6 $$v=4/{\pi }^{2}{\left({\mathrm{tan}}^{-1}{w}^{gt}/{h}^{gt}-{\mathrm{tan}}^{-1}w/h\right)}^{2},$$

where ${\rho }^{2}\left(b,{b}^{gt}\right)$ is the distance between the predicted center point and the true center point, and *w* and *h* are width and height, respectively. For this case, most of the electronic components are standard parts, and the aspect ratio is relatively stable. Therefore, the center point distance likely dominates the error more than the aspect ratio. We multiply the modulation coefficient β(0 < β < 1) before the aspect ratio term v^2⁄[(1 − Iou) + v]. The purpose is to increase the proportion of the center point distance in training and increase positioning accuracy. We determine through experiments that the value of the modulation coefficient β is 0.3, which is explained in detail in “Experimental results and discussion”. The improved loss function is

7 $${L}_{ciou}=1-IoU+{\rho }^{2}\left(b,{b}^{gt}\right)/{c}^{2}+\beta \times {v}^{2}/\left[\left(1-Iou\right)+v\right],$$

8 $$v=4/{\pi }^{2}{\left({\mathrm{tan}}^{-1}{w}^{gt}/{h}^{gt}-{\mathrm{tan}}^{-1}w/h\right)}^{2}.$$

**Figure 3:**
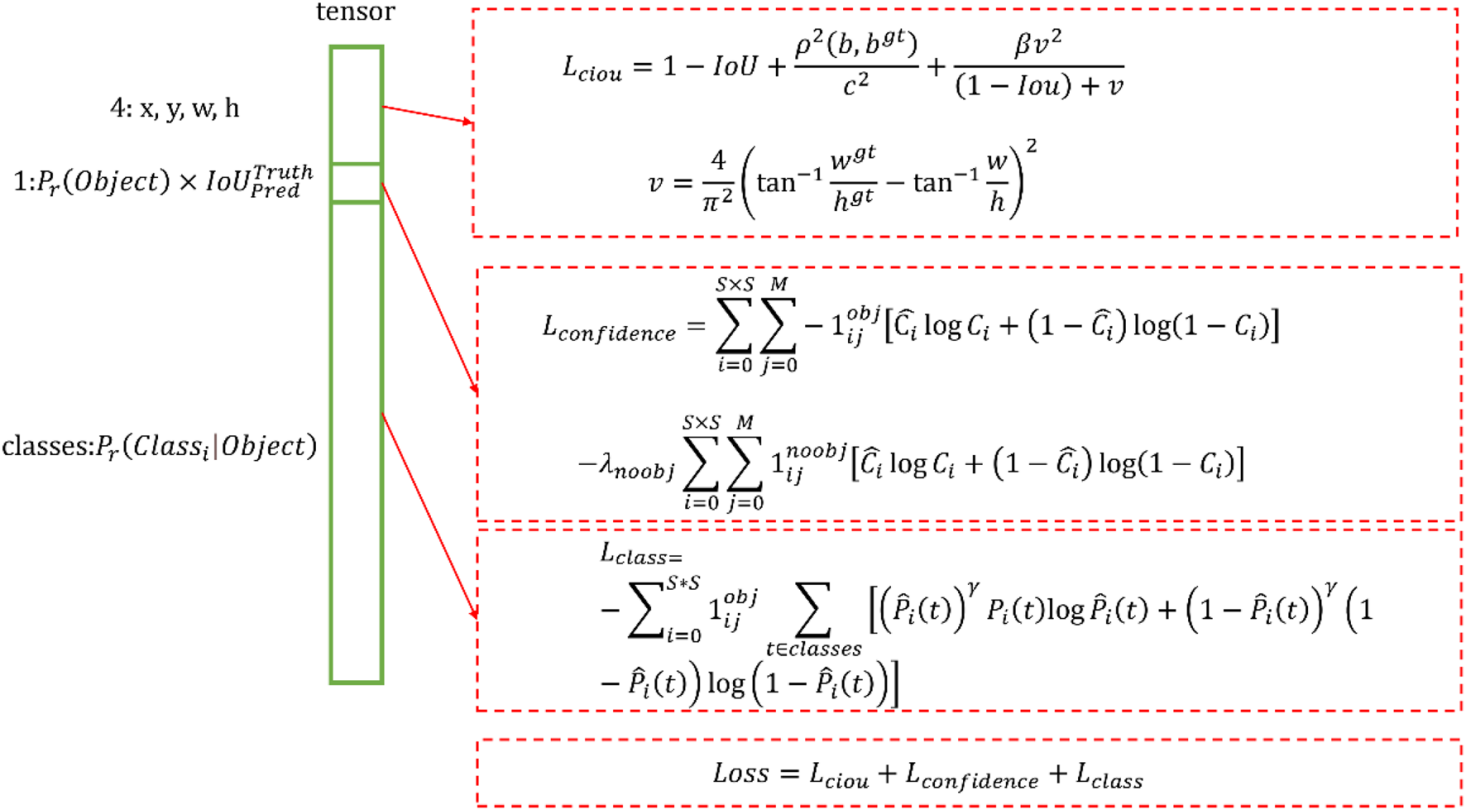
C-YOLOv4 loss function. S × S is the number of grid points, and M is the number of anchor boxes on each grid. ${1}_{ij}^{obj}$ corresponds to whether a target is present in the grid (existence is 1, otherwise it is 0), ${1}_{ij}^{noobj}$ is the opposite, and ${\lambda }_{noobj}$ is used to balance positive and negative samples. The anchor box corresponding to each grid has a confidence level ${C}_{i}$.

Combining the above modifications, we obtain the new loss function in Fig. 3. The left side of Fig. 3 is the tensor information output by the CNN, which is divided into three parts: position information, confidence, and classification information, corresponding to L_ciou, L_confidence, and L_class. These three parts of the loss function are directly added to form the final loss function. For ease of presentation, C-YOLOv4 is used in the rest of this article to represent the YOLOv4-tiny method that uses this loss function.

### Multiscale attention module

**Figure 4:**
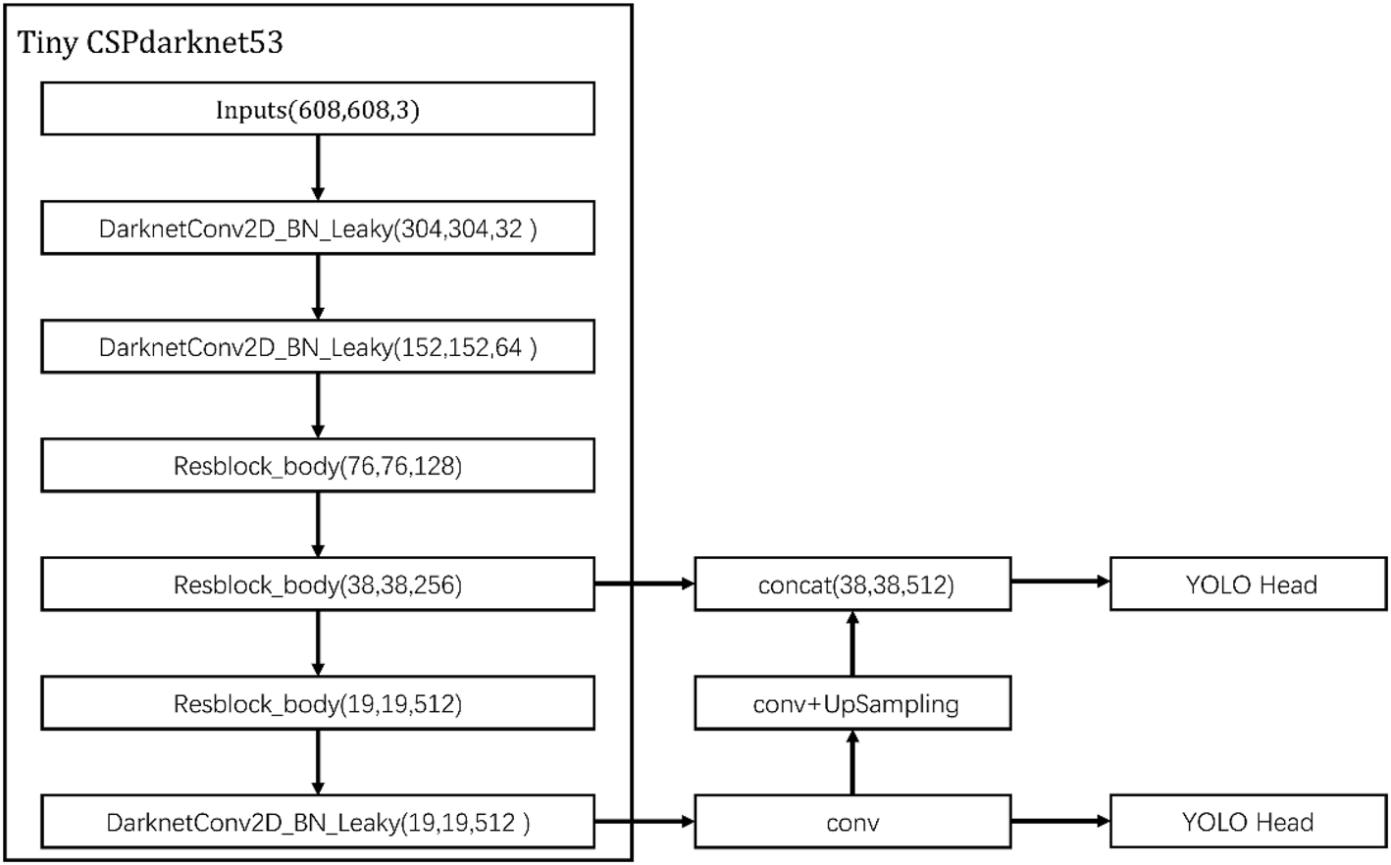
YOLOv4-tiny architecture.

Figure 4 shows the network structure of YOLOv4-tiny. The middle-level features are sent with a scale of 38 × 38, and the high-level features are sent with a scale of 19 × 19 into feature pyramid networks (FPN) for feature fusion and then into the prediction module for target detection. The FPN structure integrates the two levels to ensure the integrity of information but may cause information redundancy. Moreover, the partial error information in the extraction process of some feature layers may have a greater effect on the final results. These redundant or erroneous features must be filtered out, and the valuable features must be focused on. Therefore, we design the multiscale attention module (MAM). In this section, we discuss the attention mechanism of different scales in MAM.

**Figure 5:**
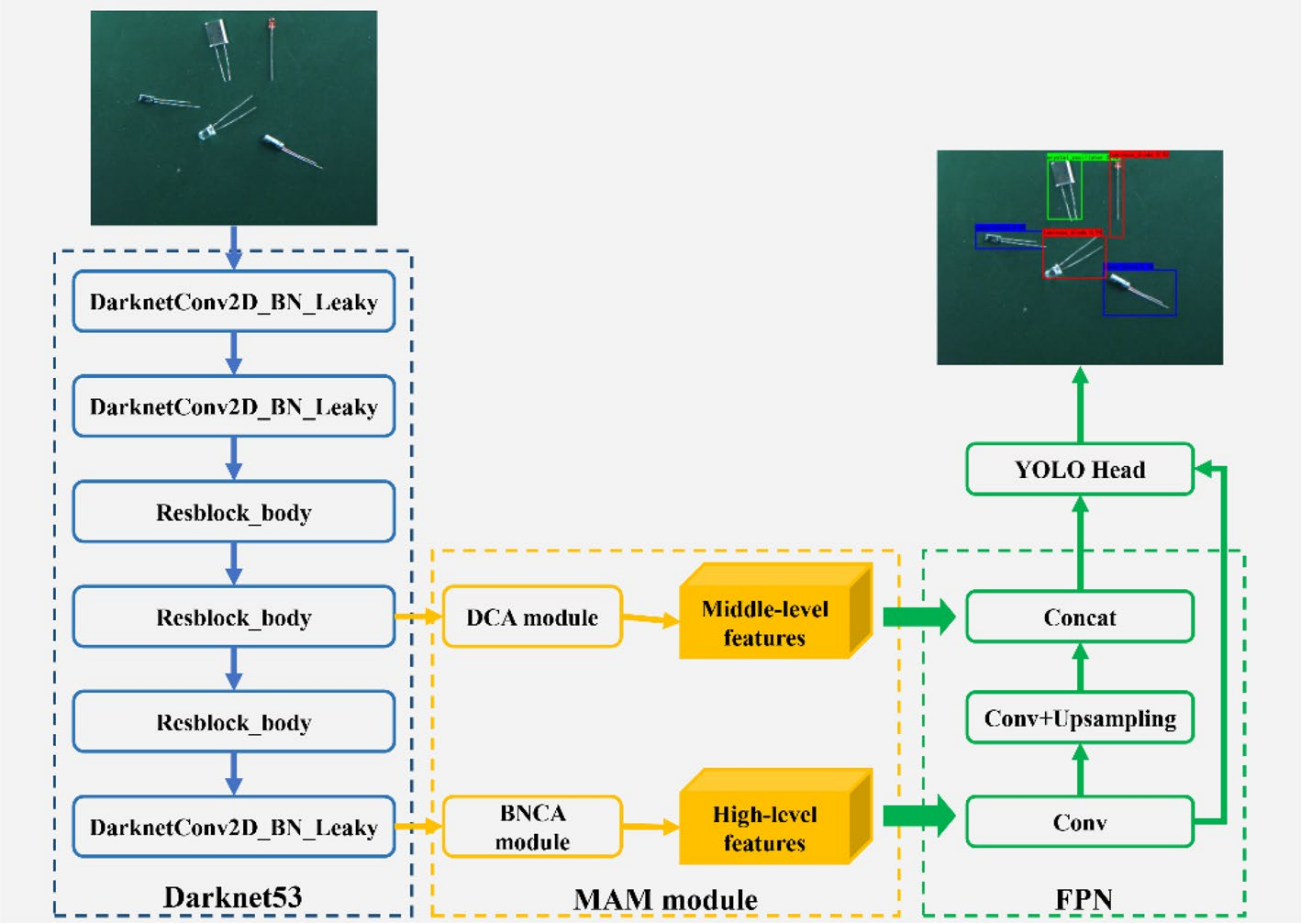
Improved YOLOv4-tiny architecture. Only the MAM is added, and the rest of the network structure remains unchanged. The structure of CSPdarknet53 adopts the original YOLOv4-tiny network structure.

Feature layers at different levels contain different amounts of semantic and spatial information. After the input image is extracted multiple times, the semantic information of the middle-level features is limited, but the spatial information increases. High-level features are rich in semantic information, and feature channels are the most complex. Therefore, we design different attention modules, namely, dual channel attention (DCA) module and batch normalization channel-wise attention (BNCA) module, for middle- and high-level features. Figure 5 shows the improved network structure, which is inspired by MAFNet^6^, the current state-of-the-art network for salient object detection. The two modules are described in detail below.

**Figure 6:**
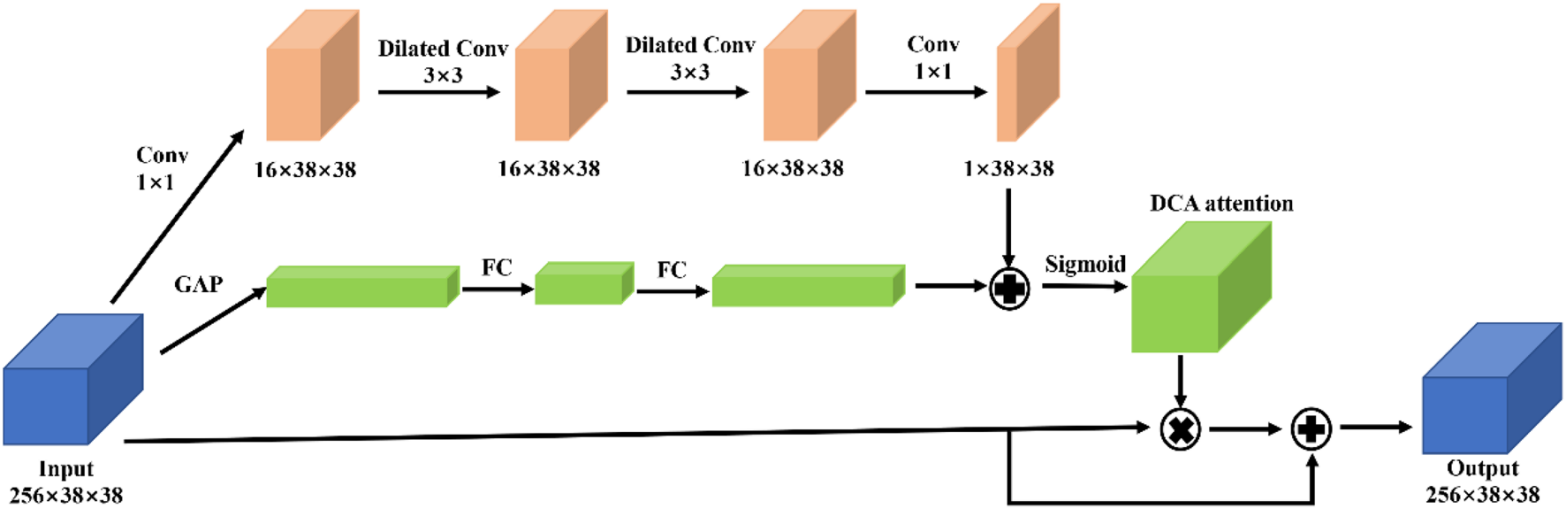
DCA module. Only the corresponding weight is applied to the original feature layer, and the size of the feature layer has not changed, so it can be directly transplanted to the YOLOv4-tiny network.

The middle feature layer contains a wealth of spatial location information and feature channel information. The DCA module is designed to make the importance of different information available through learning. Traditional convolution performs deep stacking of convolutional layers by establishing pixel relationships in the local domain, making it difficult to obtain global information. When solving the problem of semantic segmentation, the document DANet ^23^ aggregates two attention mechanism modules, which effectively enhance feature representation without adding too many parameters, and obtains the global dependency between each part. We refer to this structure, but the implementation method is different. The specific details of the DCA module are shown in Fig. 6. GAP stands for global average pooling ^24^, which can effectively obtain global information. After the GAP operation, the fully connected layer is designed twice to obtain the channel weight. In the spatial attention part, 1 × 1 ordinary convolution is used at the beginning and end to reduce the channel, and the middle part adopts the form of Dilated Conv to enhance the receptive field effectively. After obtaining the spatial and channel weights, we adopt an element-wise addition method for integration, which protects the upper layer of information while integrating. This method has been proven to be effective in BAM^25^.

**Figure 7:**
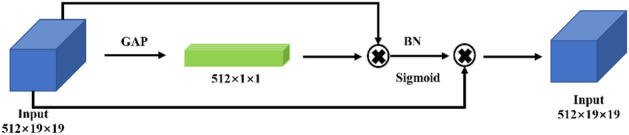
BNCA module. The attention mechanism is applied to each channel through the Global Average Pooling and sigmoid functions, and the size of the feature layer is not changed in the end.

The high-level features are obtained from iterative feature extraction by a deep neural network with the highest number of channels. For this feature layer, we use the BNCA structure shown in Fig. 7. First, GAP is used to generate channel statistics, and then two consecutive fully connected layers are used to capture the dependencies between channels. Then, it is normalized using a sigmoid function that maps to [0, 1]. Finally, the output of the block is obtained by multiplying element-wise with the original feature layer. In contrast to most channel attention, we add a batch normalization^26^ operation to improve training effect. The batch standardization method can improve the training effect, increase the model convergence speed, reduce the number of training, and alleviate over-fitting to a certain extent.

## Experimental results and discussion

### Experimental setup

In this part, we first determined the optimal values of the parameters mentioned above on the basis of experiments and then compared the proposed method with the original YOLOv4-tiny. In addition, it was compared with other current mainstream algorithms, such as Faster RCNN, SSD, EfficientDet, RefineDet, and YOLOv4. The specific experimental methods and results are described below.

#### Dataset collection and processing

**Figure 8:**
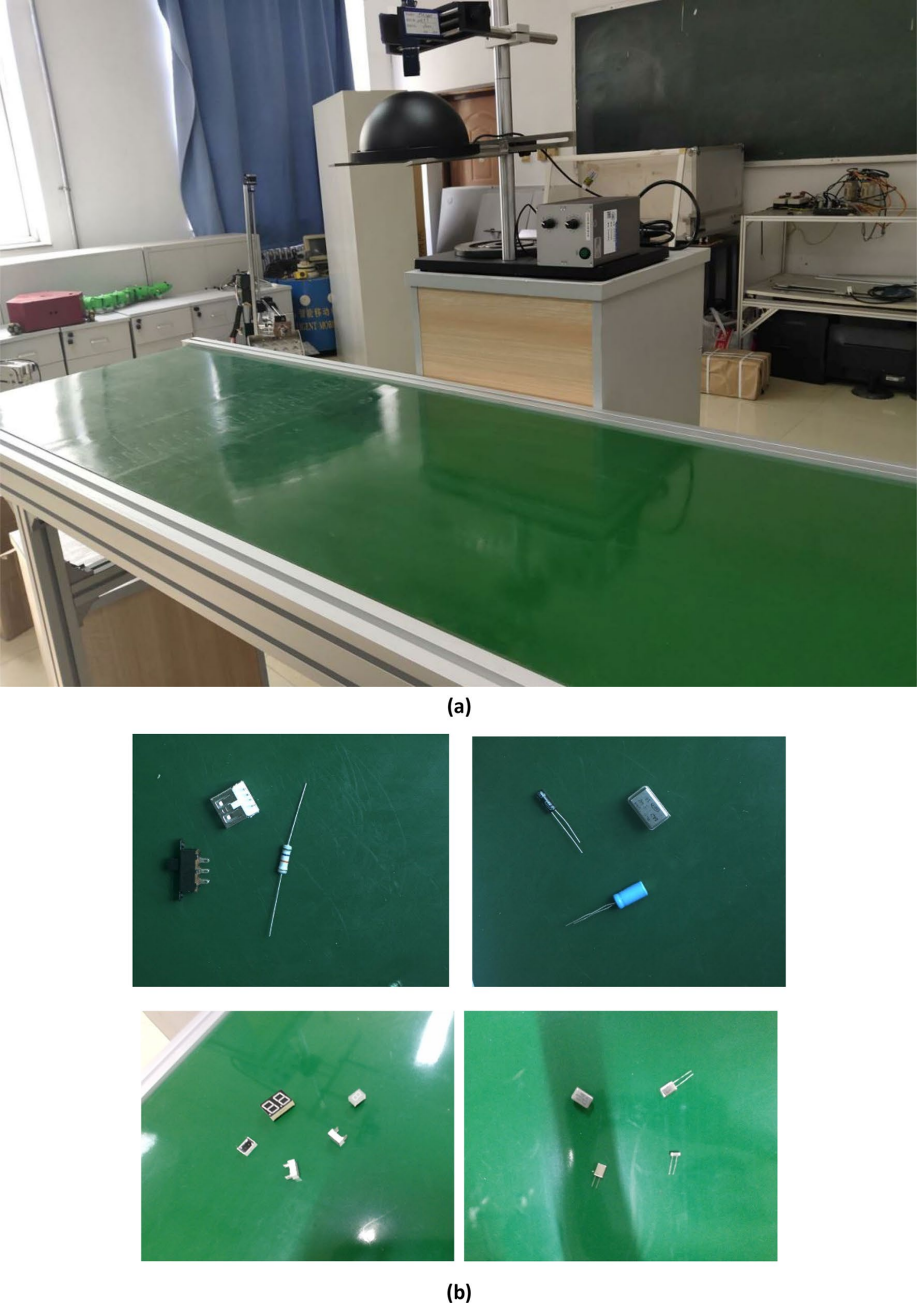
Data set collection. (**a**) A target detection platform was built to simulate an industrial environment. (**b**) The electronic components were placed and the data set was collected.

We simulated the manufacturing environment of the electronics industry and built the target detection platform shown in Fig. 8a. The industrial camera is correctly installed on the top of the pole stand, and the circular light source is below. Because the deep learning method is based on the science of a large amount of data, we have collected the data set from different angles as much as possible, and the position of the pole stand is not fixed. The CCD industrial camera mentioned in Sect. “Visual system and our visual detection tasks” was used to collect data of 20 types of electronic components placed on the conveyor belt. The list of electronic components is shown in Table 1. We collected a total of 6000 images at 11:00 a.m., and the daylight was bright at this time and the lighting source was not turned on. Since the research is aimed at manufacturing robots in the electronics industry, we often hope that robots can work 24 h a day when conditions permit. Therefore, the night situation was supplemented on the basis of the day to make system more accurate and robust. a total of 2000 images were collected at 7:30 p.m. Each image sample is created by randomly picking up multiple categories of electronic components. To increase the diversity of the dataset, conventional methods such as random flipping, shearing, and scaling were used to augment the dataset. A total of 12,000 RGB images were collected, 60% of which were used as the training set, and the verification set and the test set each accounted for 20%. Figure 8b shows part of the dataset. The upper two are images taken at night, and the lower two are images taken during the day. The size of the collected image is (1080, 1080). The YOLOv4-tiny algorithm then read the original picture and resized the picture to (608, 608) as the network input.

**Table 1:** Categories and usages of the electronic components.

Category	Sample image	Size (mm)	Aspect ratio	Function
Electric resistance		26*2.5	10.4	Resistance is a current-limiting element, usually two pins. When the resistance is connected to the circuit, it can limit the current through the branch to which it is connected
Capcitance		38*4.5	8.44	Capacitances’ positive electrode is metal foil (Al or Ta), closed to the electrolyte, which is oxide film (Al_2_O_3_ or Ta_2_O_3_), and its cathode is composed of conductive material, electrolyte (electrolyte can be liquid or solid) and other materials. Its role in the circuit is stop direct current while passing through alternating current
DC plug		18*10.5	1.71	The DC plug is widely used in various audio-visual equipment, digital cameras, toys, mobile phones, notebook computers, MP3, MP4, DV, radio tape recorders, telephones, repeaters, emergency lights, televisions, massagers, headphones, household appliances and power tools
Film capacitor		28*9	3.11	The film capacitor is a capacitor with excellent performance, and has good characteristics of no polarity, high insulation resistance, excellent frequency characteristic (wide frequency response), and small dielectric loss
Micro Mot		32*12	2.67	Micro motor refers to a motor with a diameter less than 160 mm or a rated power less than 750 mW. Micromotors are often used in control systems or transmission mechanical loads to realize electromechanical signals or energy detection, analytical operations, amplification, execution, or conversion functions
Photoresistance		28*3.5	8	The photoresistance is a special resistor made of a semiconductor material such as a sulfide or a selenide, the working principle of which is based on the internal photoelectric effect. The stronger the illumination, the less the resistance
Tantalum capacitor		18*5	3.6	The tantalum capacitor is an electrolytic capacitor with a small volume and large capacitance. It has a wide working temperature range and high specific capacity, so it is especially suitable for miniaturization
IC chip		9*7	1.29	The IC chip is an integrated circuit formed by a large number of microelectronic components (transistors, resistors, capacitors, etc.) on a plastic base to form a chip. Thus, the electronic component achieves a big step forward in the aspects of miniaturization, low power consumption and high reliability
Battery		16*7	2.29	The battery is a device that converts chemical energy into electric energy. Using the battery as the energy source, we can obtain a current with stable voltage, current, stable and power supply for a long time, and minimal influence by the outside world
Slide switch		23.5*15	1.57	The switch is the operation unit that uses electronic circuit and power electronic device to turn the circuit on and off
Luminous diode		34*3	11.33	The light-emitting diode acts as indicator lights or forms text or digital display in circuits and instruments
Quartz piezoelectric resonator		26*9.5	2.73	The quartz crystal resonator has the characteristics of stable and good anti-interference performance
Ceramics piezoelectric resonator		15*13	1.15	The ceramic crystal resonator is a frequency component of the piezoelectric ceramic class. Its main function is to convert the electric energy in the circuit into mechanical energy to produce a predetermined stable frequency
7 segment LED		20*12	1.67	The nixie light is an electronic device that can display digital and other information. Most cathodes of the nixie light are in the shape of numbers. The tube is filled with low-pressure gas, most of which is neon. To charge a cathode, the nixie light emits a colored light
Inductors		22*3.5	6.29	The inductor is a group of coaxial turns made of enameled wire, yarn-wrapped wire, or plastic wire in series on an insulated skeleton or magnetic core. Its main function is to isolate the AC signal, filter or form resonant circuit with capacitor, resistor, and so on
Fuse		17*2.5	6.8	The fuse acts as overload protection and protects the safe operation of the circuit
Crystal triode		25*7.5	3.33	The crystal triode transistor is a semiconductor device that controls the current. Its function is to amplify the weak signals into electric signals with a large amplitude value, and it is also used as a contactless switch
Field-effect transistor		28*9	3.11	The field-effect transistor is a voltage-controlled semiconductor device. It has the advantages of high input resistance (107–1015 Ω), low noise, low power consumption, large dynamic range, easy integration, no secondary breakdown phenomenon, and wide safe working area
USB connector		19*16	1.19	Universal Serial Bus (USB) is a serial bus standard and a technical specification for input and output interfaces. It is widely used in information communication products such as personal computers and mobile devices, and extends to photographic equipment and digital TVs (set-top boxes), game consoles, and other related fields
Pin header		25*10	2.5	Pin headers are generally widely used in the connection of PCB boards and are known as universal connectors.It generally paired with headers, wire ends, and other connectors

#### Evaluation metrics

We used mAP and FPS to evaluate the improvement methods we proposed. The following is a detailed introduction to the indicators.

In the multilabel image classification task, a picture has more than one label, that is, each image may have different categories of targets. Therefore, the evaluation cannot use the common single-label image classification standard. The object detection task uses a similar method to information retrieval—mAP, which is the average value of average precision in each category. Average precision is defined as the area enclosed by the accuracy and recall curve and the coordinate axis. The accuracy rate and recall rate are calculated as follows:

- Precision: $$TP/\left(TP+FP\right)$$
- Recall: $$TP/\left(TP+FN\right)$$

True positive (TP) means that the model predicts a positive sample, and the actual prediction is correct; false positive (FP) means that the model predicts a positive sample, and the detection is wrong; and false negative (FN) means that the model predicts a negative sample, and the detection is wrong.

FPS is currently a common indicator for evaluating model efficiency. In two-dimensional image detection, it is defined as the number of pictures that can be processed per second.

#### Implementation details

We used Intel Core i7-8700 CPU, NVIDIA TITAN Xp GPU, 32 GB RAM to build the hardware system. The operating system is Windows 10; TensorFlow 1.13.1 and Keras 2.1.5 deep learning environment are installed. The following introduces the setting of the basic parameters of YOLOv4-tiny.

Batch_size is the number of images sent to training at a time. A larger batch_size can make model training more stable, but it increases the calculation amount. Considering the computing power of the graphics card we gused, we chose 16 as the batch_size.

Learning rate is the speed of iterative training of the model, and the correct setting of the learning rate can make the loss curve smoother. The initial learning rate was set to $1\times {e}^{-4}$, and the minimum decay learning rate was $1\times {e}^{-6}$. The adaptive moment estimation (Adam) optimizer was used for optimization. We performed cluster analysis on the samples and selected six anchors with sizes (10, 14), (23, 27), (37, 58), (81, 82), (135, 169), and (344, 319). A total of 100 epochs were trained.

### Results and analysis

The original loss function was replaced with the formula shown in Fig. 2, and the value of the γ and β modulation parameter in the formula was determined through experiments. The test set accuracy is summarized in Table 2. The results show that with the increase of γ, the detection accuracy of electronic components first increases and then decreases, and the effect is best when γ is set to 1.5. With the gradual decrease of β, the slight limitation on the aspect ratio calculation cannot play a vital role, and the excessive limitation on the aspect ratio calculation leads to an imbalance in the loss function. The best effect is achieved when β is set to 0.3. In the case where the two parameters act simultaneously, it can still improve the accuracy. We finally determined γ and β to be 1.5 and 0.3, respectively.

**Table 2:** The effect of γ and β on accuracy.

Map	β									
	None	0.9	0.8	0.7	0.6	0.5	0.4	0.3	0.2	0.1
**γ**										
None	93.94	93.25	93.92	93.19	94.10	94.32	94.56	94.71	94.11	90.46
1.1	94.25	94.31	93.86	93.95	94.58	94.67	94.87	94.92	94.51	91.56
1.2	93.27	93.48	94.15	93.15	93.77	94.06	94.22	94.36	93.82	91.17
1.3	94.19	94.35	94.18	94.08	94.37	94.72	95.02	95.21	94.31	92.36
1.4	94.54	94.23	95.33	94.42	94.88	95.13	95.25	95.33	94.51	92.87
1.5	95.27	94.86	95.62	95.21	95.52	95.79	95.85	**95.92**	95.83	94.19
1.6	93.56	93.21	93.79	93.05	94.02	94.29	94.33	94.89	94.01	92.48
1.7	94.71	95.11	95.18	94.56	94.87	94.95	95.17	95.36	94.85	92.84
1.8	94.63	94.57	94.68	94.45	94.75	94.81	95.11	95.23	94.77	92.63
1.9	92.46	93.04	93.52	92.37	93.19	93.55	93.82	94.07	93.84	91.27

Maximum values are in bold.

**Figure 9:**
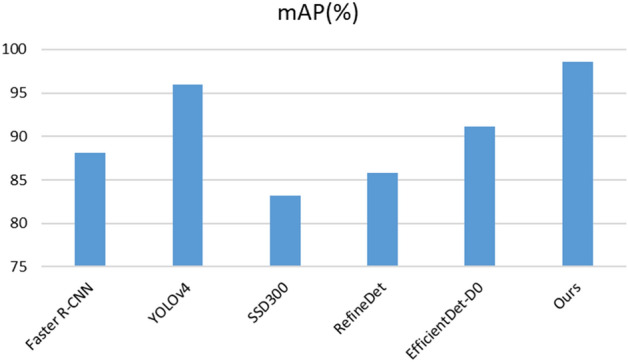
Comparison of accuracy with other advanced algorithms.

**Figure 10:**
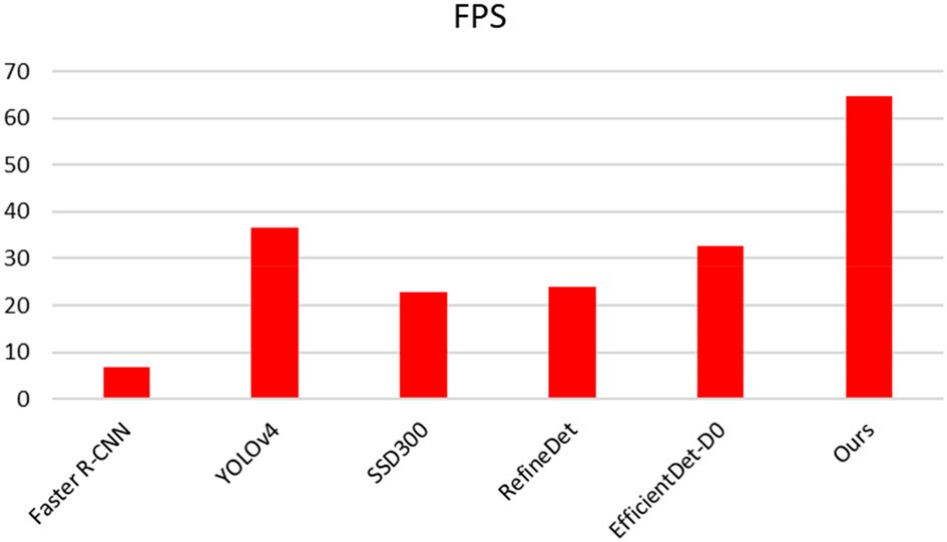
Comparison of efficiency with other advanced algorithms.

To ensure the authenticity of the results, MAM was re-deployed on the basis of the original YOLOv4-tiny, and the training parameters were completely consistent. The accuracy and speed and the total network parameters were calculated. The two methods were simultaneously deployed in YOLOv4-tiny. DCA and BNCA refer to the experimental results without C-YOLOv4 to reflect the effect of different methods deployed in the original algorithm. The results are shown in Table 3. We deployed other current state-of-the-art algorithms to this case to verify the effectiveness and superiority of the method. The results are shown in Figs. 9 and 10.

**Table 3:** Evaluation results on the test set.

Method	Parameters (M)	FLOPs (B)	mAP (%)	FPS
YOLOv4-tiny	5.88	6.9	93.94	67
C-YOLOv4	5.88	6.9	95.92	**67.6**
DCA	6.11	7.2	95.37	64.7
BNCA	5.92	7.0	96.12	65
C-YOLOv4 + MAM	6.15	7.4	**98.6**	64.8

Maximum values are in bold.

**Figure 11:**
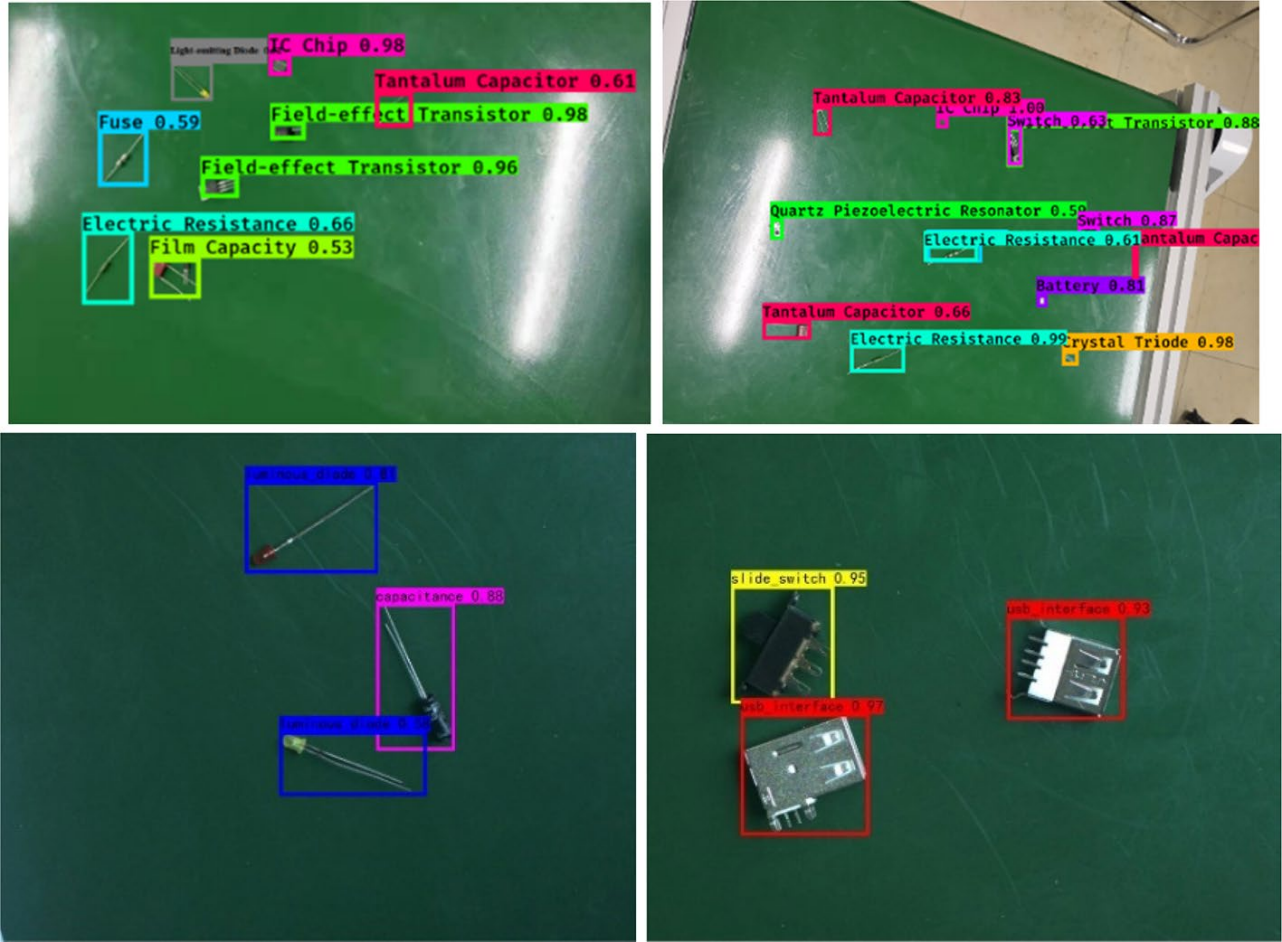
Inspection results of electronic components.

Table 3 shows that the accuracy of the two proposed methods is significantly improved in comparison with that of the original algorithm. The accuracy of the two methods increased from 93.94 to 98.6% under the combined use of the two methods. In terms of computational efficiency, compared with the original algorithm, MAM adds a parameter of 0.27 M, resulting in a slight decrease in speed, but the accuracy is significantly improved at the expense of a small amount of speed. The frame rate of the CCD industrial camera used by the robot in this case was 24 FPS, which meets the needs of real-time detection. In Figs. 9 and 10, we compared the performance of other advanced target detection algorithms on the electronic component dataset. The results show that our method has the best accuracy and speed. We used the camera to collect and predict the parts placed on the industrial conveyor belt in real time, and some of the results are shown in Fig. 11. The upper two are images taken at day, and the lower two are images taken during the night.

## Conclusion

This study explored the detection of electronic components by industrial robots in the electronics industry. This detection task is challenging because robots’ vision guidance requires high accuracy and real-time performance, the electronic components are small, and categories are difficult to distinguish. On the basis of the YOLOv4-tiny detector, we proposed two optimization methods. First, the loss function and network structure were modified to make the algorithm adapt to the detection of electronic components. In addition, we designed a DCA module for the middle-level features and added an attention mechanism from the two channels. The BNCA module was designed for high-level features, the linear and nonlinear relationship between each channel was obtained, and batch standardization was used to alleviate overfitting. The modification of the cross-entropy function alleviates the difficulty of classifying small-sized parts to a certain extent. The addition of the attention mechanism allows the neural network to focus on more valuable features, thus solving the problem of the small proportion of electronic components in the picture. We created a dataset of electronic components and verified the effectiveness of the method through experiments. The results showed that our method achieves significantly improved detection accuracy in comparison with the original algorithm when the speed is only slightly reduced. Moreover, our method meets the requirements of real-time detection in an industrial environment. In the future, we will focus on further lightening the model and reducing the calculation amount to deploy it to embedded computing devices.
